# Practices related to assessment of sedation, analgesia and *delirium* among critical care pediatricians in Brazil

**DOI:** 10.31744/einstein_journal/2020AO5168

**Published:** 2020-01-22

**Authors:** José Colleti, Orlei Ribeiro de Araujo, Alice Barone de Andrade, Werther Brunow de Carvalho

**Affiliations:** 1 Hospital Santa Catarina São PauloSP Brazil Hospital Santa Catarina, São Paulo, SP, Brazil.; 2 Instituto de Oncologia Pediátrica São PauloSP Brazil Instituto de Oncologia Pediátrica, Grupo de Apoio ao Adolescente e à Criança com Câncer (GRAAC), São Paulo, SP, Brazil.; 3 Hospital das Clínicas Faculdade de Medicina Universidade de São Paulo São PauloSP Brazil Instituto da Criança, Hospital das Clínicas, Faculdade de Medicina, Universidade de São Paulo, São Paulo, SP, Brazil.

**Keywords:** Deep sedation, Analgesia, Delirium, Critical care, Child, Surveys and questionnaires, Brazil

## Abstract

**Objective:**

To understand the use of tools, protocols and comfort measures related to sedation/analgesia, and to screen the occurrence of *delirium* in pediatric intensive care units.

**Methods:**

A survey with 14 questions was distributed by e-mail to Brazilian critical care pediatricians. Eight questions addressed physician and hospital demographics, and six inquired practices to assess sedation, analgesia, and *delirium* in pediatric intensive care units.

**Results:**

Of 373 questionnaires sent, 61 were answered (16.3%). The majority of physicians were practicing in the Southeast region (57.2%). Of these, 46.5% worked at public hospitals, 28.6% of which under direct state administration. Of respondents, 57.1% used formal protocols for sedation and analgesia, and the Ramsay scale was the most frequently employed (52.5%). *Delirium* screening scores were not used by 48.2% of physicians. The Cornell Assessment of Pediatric *Delirium* was the score most often used (23.2%). The majority (85.7%) of physicians did not practice daily sedation interruption, and only 23.2% used non-pharmacological measures for patient comfort frequently, with varied participation of parents in the process.

**Conclusion:**

This study highlights the heterogeneity of practices for assessment of sedation/analgesia and lack of detection of *delirium* among critical care pediatricians in Brazil.

## INTRODUCTION

Sedation and analgesia are important and necessary components of care for the majority of patients admitted to a pediatric intensive care unit (PICU), particularly patients requiring mechanical ventilation (MV).^1 , 2^ Major indications include control of pain, anxiety and agitation; induction of amnesia; facilitation of MV (reduction of asynchrony); prevention of endotracheal dislodgement; and reduction of cell metabolism.^1 , 2^

The adverse impact of inefficient sedation and analgesia practices at the PICU has become the focus of attention for researchers and clinicians, alongside concerns generated by the use of too light or too deep sedation levels.^3^ Both inadequately light and excessively deep sedation have the potential to produce safety problems for patients, and effects on the duration of MV, on hospital length of stay and costs.^3^ Consequences of prolonged use of sedative and analgesic drugs at the PICU include changes in the central nervous system, gastrointestinal disturbances and sympathetic hyperactivity. Children under inadequate sedation and/or analgesia are at risk of losing vascular access, accidental tracheal extubating, falls, post-traumatic stress disorder and changes in neurodevelopment.^4^

The distinction between pain, anxiety and *delirium* in children can be challenging, in part due to communication barriers linked to development, and to the presence of severe diseases. Besides, the most commonly used medications (opioids and benzodiazepines) can cause hemodynamic and respiratory instability, prolonged MV, abstinence symptoms, *delirium* , nosocomial infection and critical illness neuromyopathy.^1 , 4^

Our assumption was of the occurrence of high variability in sedation and analgesia approaches for the critically ill child admitted to the PICU, and that sleep promotion and detection of *delirium* are not routinely implemented.

## OBJECTIVE

The study aimed to understand the use of tools, protocols and comfort measures related to sedation/analgesia, in addition to screening for the occurrence of *delirium* in pediatric intensive care units.

## METHODS

### Development of the survey

The authors designed a questionnaire addressing practices related to sedation and analgesia at PICU. in Brazil, based on a literature review. Toward that end, the authors searched the keywords “ *sedation and analgesia and children* ”, “ *delirium and children* ”, “ *sedation and (guidelines or protocol) and children* ”, “ *analgesia and (guidelines or protocol) children* ”, at the PubMed^®^, EMBASE and Scientific Electronic Library Online (SciELO) databases for the period January 2010 to January 2017.

The survey was tested and validated with five critical care pediatricians from distinct Brazilian centers, using a questionnaire as a clinically sensitive tool. The feedback for each question was analyzed considering scope and writing, presence of reductant or inappropriate items, and if the questionnaire complied with the objectives of the survey. The answers of the pilot phase were not included in the results reported in the study. The questionnaire was sent to the critical care pediatricians enrolled in the *Associação de Medicina Intensiva Brasileira -* AMIB [Brazilian Critical Care Association] database. The study was approved by the Research and Ethics Committee of *Associação Congregação de Santa Catarina* , resolution 3,077,035; CAAE: 02880918.1.0000.9007. E-mail data were collected from June to November, 2017.

### Characteristics of the survey

The questionnaire consisted of 14 multiple choice questions. The first six questions addressed demographic aspects of the physicians and places of work. The remaining eight questions focused specifically on routine sedation and analgesia practices, protocol adherence and detection of *delirium* by critical care pediatricians at the PICU of their practice.

### Statistical analysis

The descriptive analysis of data collected consisted of calculation of simple frequencies and proportions using software R, version 3.5.0.

## RESULTS

The sample consisted of 61 critical care pediatricians who answered the questionnaire, out of 373 e-mails sent (answer rate of 16.3%). After initial analysis of answers, five physicians were excluded because they worked exclusively at a neonatal ICU. Table 1 shows demographic and descriptive data of participants’ workplace. Most of the critical care pediatricians who answered the questionnaire were from the southern region of Brazil (57.2%), and 73.2% practiced at a PICU with a maximum number of 15 beds. From the management point of view, 46.5% practiced at public hospitals, and of those 28.6% of hospitals were under direct management of the government and 17.9% under indirect management, and 41.1% of intensive care physicians practiced at private hospitals. Of the physicians participating in the survey, 64.3% practiced at hospitals with residency programs in pediatric intensive care medicine.

**Table 1:** Descriptive and demographic characteristics of hospitals and pediatricians who took part in the survey

Characteristics	
Region	
North	1.8
Northeast	14.3
Center West	14.3
Southeast	57.0
South	12.6
Type of ICU	
Pediatric ICU	41 (73.2)
Mixed ICU (pediatric + neonatal)	6 (10.7)
Cardiology ICU	3 (5.4)
Others	6 (10.7)
Number of beds	
<10	19 (33.9)
10-15	22 (39.3)
16-20	10 (17.9)
>20	5 (8.9)
Hospital management	
Public (direct management)	16 (28.6)
Public (indirect management)	10 (17.9)
Private	23 (41.1)
Mixed	5 (8.9)
Did not answer	2 (3.5)
Medical residency program	
Yes	36 (64.3)
No	20 (35.7)
Patients on MV at ICU, %	
<25	8 (14.3)
25-50	22 (39.3)
51-75	18 (32.1)
>75	8 (14.3)
Time of medical residency, years	
1	6 (10.7)
2	8 (14.3)
3	13 (23.2)
4	23 (41.1)
>4	6 (10.7)
Time working at ICU, years	
<3	12 (21.4)
3-6	11 (19.7)
6-9	5 (8.9)
9-12	8 (14.3)
>12	20 (35.7)

Results expressed as % or n (%).

ICU: intensive care unit; MV: mechanical ventilation.

Table 1 describes the time participants underwent training. Most of them (51.8%) spent 4 or more years in medical residency training programs. Regarding the time of medical practice in a PICU, we observed a bimodal distribution with extreme values, since 41% of participants had 6 or less years of professional experience, while 12 and more years of experience were reported by 35.7% of participants.

Because the use of sedation and analgesia is mainly related to patients requiring MV, one question was related to the daily mean percentage of patients under their care that required MV. Answers were that 39.3% of participants assisted from 25 to 50% of patients on MV, and 32.1% of participants from 50 to 75% of patients on MV, totaling up 71.4% of participants with 25% to 75% of patients on MV daily.

**Figure 1:**
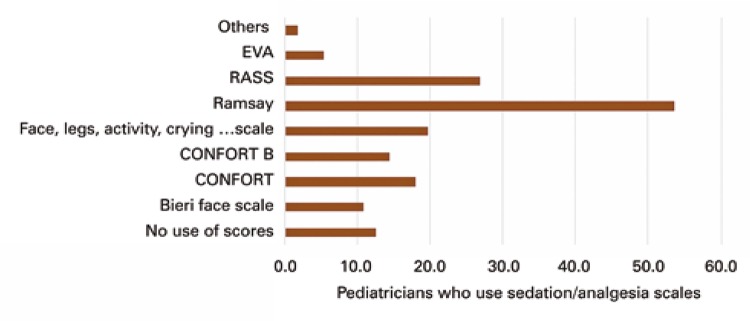
Sedation and analgesia assessment scales used by survey participants VAS: visual analog scale; RASS: Richmond agitation-sedation scale.

**Figure 2:**
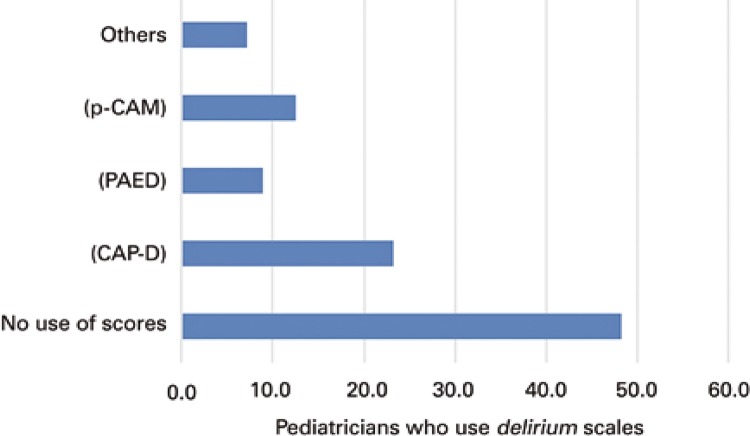
*Delirium* assessment scales used by survey participants p-CAM: Pediatric Confusion Assessment Method for the Intensive Care Unit; PAED: Pediatric Anesthesia Emergence *Delirium* Scale; CAP-D: Cornell Assessment of Pediatric *Delirium.*

Most participants (87.5%) used sedation and analgesia scales at their units; however only 57.1% of PICU had a formal protocol. The most commonly used scales were the Ramsay sedation scale (53.6%) and the Richmond Agitation-Sedation Scale (RASS) (26.8%) ( Figure 1 ). In the majority of units where protocols were employed, they were managed by physicians (53.6%). On the other hand, 48.2% of physicians did not use scales for detecting *delirium* , and when a scale was employed, the Cornell Assessment of Pediatric *Delirium* (CAP-D) was the most frequently used (23.2%) ( Figure 2 ).

**Figure 3:**
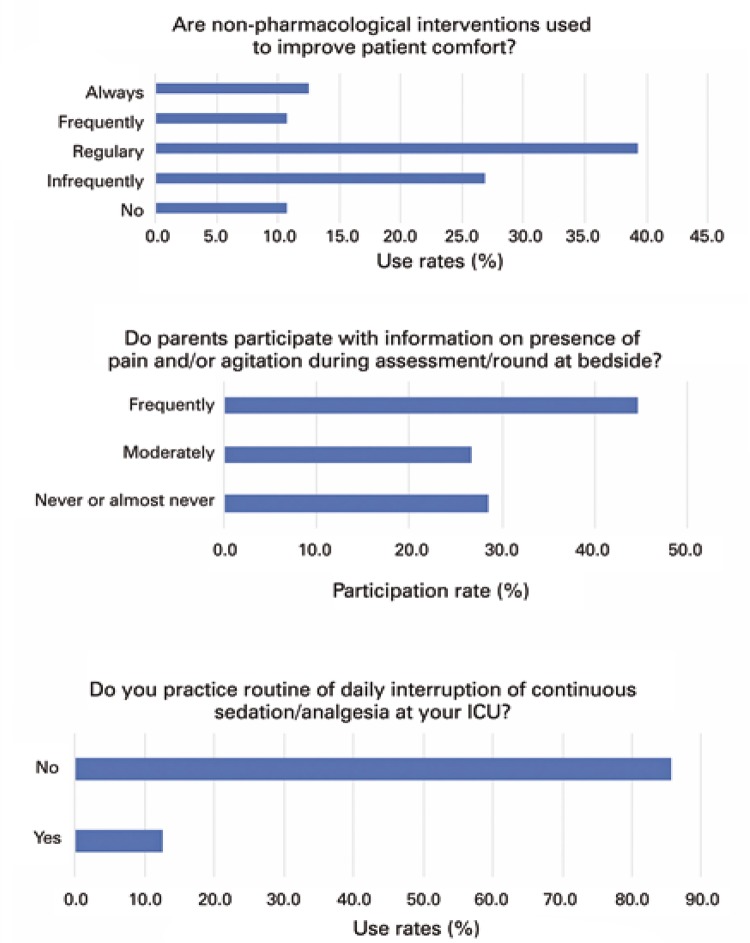
Other interventions associated with daily sedation, analgesia and *delirium* practices ICU: intensive care unit.

Regarding additional practices assessed in the study, non-pharmacological interventions were reported as always or frequently used only by 23.2% of participants. The item describing the participation of parents informing the presence of pain or agitation showed heterogeneous results, and 44.6% of survey respondents answered that there was an effective participation of parents in the process ( Figure 3 ). The majority of participants (85.7%) did not comply with the practice of daily interruption of sedation in patients on MV at the PICU ( Figure 3 ).

## DISCUSSION

This is the first study to investigate the use of clinical tools to assess sedation, analgesia and prevention of *delirium* by critical care pediatricians in Brazil. The study showed that pain and sedation are routinely assessed during clinical practice of critical care pediatricians. They reported following sedation and analgesia guidelines and protocols at their units. A minority (12.5%) was not familiar with scales to assess sedation and analgesia. The data revealed satisfactory adherence to the utilization of tools to assess patient sedation and analgesia. As a means of comparison of the same practice in other countries, Garcia Guerra et al.,^5^ reported that 84% of critical care pediatricians in Canada used sedation and analgesia scales at their units, and the most widely used scales were COMFORT (41%) and COMFORT-B (15%). Kudchadkar et al.,^6^ in an international study (although 70% of participants were from North America), showed that, even if 70% of questionnaire respondents stated having scales for sedation assessment in their PICU, only 42% used them routinely to establish patient care goals. One of the main objectives for designing protocols to assess sedation and analgesia is the use of clinical tools validated for pediatric use that enable the rational use of pharmacological and non-pharmacological measures aimed at patient comfort.

As to assessment of *delirium* by respondents, 51.8% used some kind of assessment tool, and the CAP-D was the most used tool. The finding highlights the presence of an important gap in patient assessment amongst survey participants. The study of Kudchadkar et al.,^6^ showed that 71% of respondents reported that there was no routine triage for *delirium* at their units, and only 2% reported that *delirium* tracking was performed in all children at least once every shift.^6^ Assessment of delirium is also uncommon at PICU in Canada.^5^ *Delirium* has been increasingly acknowledged as a frequent complication in intensive care and clearly associated with negative outcomes, including mortality. Guidelines for sedation in adults recommend monitoring it routinely.^7 , 8^ However, there is a lack of high-quality studies in pediatrics, and the current prevalence estimates range from 4.5% to 28%.^9^ Additionally, it is not clear which is the best approach for prevention, detection and management of pediatric *delirium* . However, any treatment strategy depends on routine monitoring and identification as its first essential step. Unfortunately, symptoms of pediatric *delirium* are frequently treated with additional sedation, establishing a vicious cycle that contributes to increase in morbidity and mortality.^10^

The practice of daily interruption of sedation and analgesia was not observed at the majority of PICU. It was more commonly practiced for adult patients, with no evidence of benefits of daily interruption in pediatric sedation. A multicenter randomized controlled study showed that daily interruption of sedation in children on MV associated with the use of sedation protocols, has not improved clinical outcomes and was associated with increase in mortality when compared only to use of sedation protocols.^11^ On the other hand, Zimmerman et al., in a letter to the editor, criticized the publication and suggested that further studies focusing on the daily interruption of sedation must be performed using a multidisciplinary approach.^12^ There is an open field for studies addressing the issue in pediatrics.

Albeit the wide range of answers, parents had some participation in the management of patient´s pain and agitation in our sample. More and more, parents are actively participating in decisions concerning children admitted to PICU. There are few quality studies that could be able to show the possible benefits of growing participation in pain and *delirium* management.

The present study showed that non-pharmacological comfort measures were commonly used by the sample studied. Promoting an environment with adequate lighting and sound, aiming to avoid stress and facilitate sleep, is important in an intensive care unit. In a systematic review, Kudchadkar et al.,^13^ stated that “although these preventive and therapeutic measures can be considered of straightforward and low-cost implementation, a large cultural change must yet happen in the pediatric intensive care community”.^13^ Scientific and clinical evidence is essential to demonstrate that sleep optimization of critically ill children can reduce morbidity, by means of decreasing sedative medications, neuro-inflammation and hospital length of stay. Other additional non-pharmacological interventions can be implemented, such as use of pacifier, music, television and electronic devices with games (tablets), among others.

The importance of the initiative ICU Liberation from the Society of Critical Care Medicine (SCCM), aiming to free ICU patients from the adverse effects of pain, agitation and *delirium* ^14^ must be highlighted. The ICU Liberation guidelines and the set of ABCDEF measures are vital resources to assess, treat and prevent pain, agitation and *delirium* .^14 , 15^ The initiative also focuses on strategies of early mobilization, that can help to reduce the risks of long-term consequences of ICU admissions.

This study has limitations. The response rate was low (16.3%) from a small medical specialty group when compared to adult intensive care physicians, reducing the statistical power of the study. The sample has a selection bias, for only critical care pediatricians registered at the AMIB were invited to participate, regardless of having the specialist title, and may have not represented the overall population of pediatric intensive care physicians in Brazil. The major part of respondents was from the Southeast region of Brazil, mostly from the State of São Paulo, discouraging a nationwide generalization of the results.

## CONCLUSION

The present study highlights the heterogeneity of sedation/analgesia assessment practices and the lack of tracking of *delirium* by a sample of critical care pediatricians in Brazil. There are several opportunities for future studies, quality improvement, design of protocols and therapeutic interventions for pediatric populations undergoing active neurocognitive development.
