# Supplementary material for: Practices related to assessment of sedation, analgesia and delirium among critical care pediatricians in Brazil
Source: Einstein (Sao Paulo). 2020 Jan 22;18:eAO5168. doi: 10.31744/einstein_journal/2020AO5168 (PMC6980295; doi:10.31744/einstein_journal/2020AO5168)
Supplement: Translation

# Translation: Práticas relacionadas à avaliação de sedação, analgesia e *delirium* entre pediatras intensivistas no Brasil

**Authors:** José Colleti, Orlei Ribeiro de Araujo, Alice Barone de Andrade, Werther Brunow de Carvalho

**Affiliations:** 1 Brasil Hospital Santa Catarina, São Paulo, SP, Brasil.; 2 Brasil Instituto de Oncologia Pediátrica, Grupo de Apoio ao Adolescente e à Criança com Câncer (GRAAC), São Paulo, SP, Brasil.; 3 Brasil Instituto da Criança, Hospital das Clínicas, Faculdade de Medicina, Universidade de São Paulo, São Paulo, SP, Brasil.

**Keywords:** Sedação profunda, Analgesia, Delírio, Cuidados críticos, Criança, Inquéritos e questionários, Brasil

## Abstract

**Objetivo:**

Compreender o uso de ferramentas, protocolos e medidas de conforto relacionadas à sedação/analgesia, além de rastrear a presença de *delirium* em unidades de terapia intensiva pediátricas.

**Métodos:**

Um inquérito com 14 questões foi distribuído, por meio de correio eletrônico, para médicos pediatras intensivistas brasileiros. Oito questões eram sobre os dados demográficos dos médicos e dos hospitais, e seis questões eram sobre as práticas na avaliação da sedação, analgesia e *delirium* em unidades de terapia intensiva pediátrica.

**Resultados:**

Responderam ao inquérito 61 médicos dos 373 e-mails enviados (taxa de resposta de 16,3%). A maioria dos médicos era da Região Sudeste (57,2%) e 46,5% trabalhavam em hospitais públicos, sendo 28,6% sob administração direta do Estado. Dos respondedores, 57,1% utilizavam protocolos formais de sedação e analgesia, sendo a escala de Ramsay a mais utilizada (52,5%). Não utilizavam escores de rastreamento de *delirium* 48,2% dos médicos, e o *Cornell Asssessment of Pediatric Delirium* (23,2%) foi o mais utilizado. A maioria (85,7%) dos médicos não utilizou a prática da interrupção diária da sedação, e apenas 23,2% utilizavam medidas não farmacológicas para o conforto do paciente com frequência, com a participação heterogênea dos pais nesse processo.

**Conclusão:**

Este estudo destaca a heterogeneidade nas práticas de avaliação da sedação/analgesia e insuficiência de rastreamento de *delirium* entre os intensivistas pediátricos brasileiros.

## INTRODUÇÃO

Sedação e analgesia são componentes importantes e necessários no cuidado da maioria dos pacientes internados em unidades de terapia intensiva (UTI) pediátrica, especialmente aqueles que necessitam de ventilação mecânica (VM).^1 , 2^ As principais indicações para seu uso incluem redução da dor, ansiedade e agitação do paciente; induzir amnésia; facilitar a VM (redução da assincronia); prevenir o deslocamento de tubos intratraqueais; e diminuir o metabolismo celular.^1 , 2^

O impacto adverso das práticas inadequadas de sedoanalgesia nas UTI pediátricas tornou-se cada vez mais foco para pesquisadores e clínicos, associando-se com preocupações relacionadas à hipossedação e hipersedação.^3^ Tanto a hipossedação quanto a hipersedação têm potencial para ocasionar problemas de segurança do paciente e impacto na duração da VM, no tempo de permanência e nos custos hospitalares.^3^ As consequências do uso prolongado de agentes sedativos e analgésicos na UTI pediátricas incluem alterações do sistema nervoso central, distúrbios gastrintestinais e hiperatividade simpática. Crianças com sedação e/ou analgesia inadequadas apresentam risco de perda de acesso vascular, extubação traqueal não planejada, queda, transtorno de estresse pós-traumático e alterações no neurodesenvolvimento.^4^

Em crianças, a distinção entre dor, ansiedade e *delirium* pode ser desafiadora, em parte devido às limitações de comunicação relacionadas ao desenvolvimento e à presença de doenças graves. Além disso, os medicamentos mais utilizados (opioides e benzodiazepínicos) podem provocar risco de instabilidade hemodinâmica e respiratória, VM prolongada, sintomas de abstinência, *delirium* , infecção hospitalar e neuromiopatia associada à doença grave.^1 , 4^

A hipótese é a de que existe uma variabilidade significativa nas abordagens de sedoanalgesia na criança criticamente enferma internada em UTI pediátrica, sendo que a promoção do sono e o rastreamento de *delirium* não são rotineiramente implementados.

## OBJETIVO

Compreender o uso de ferramentas, protocolos e medidas de conforto relacionadas à sedação/analgesia, além de rastrear a presença de *delirium* em unidades de terapia intensiva pediátricas.

## MÉTODOS

### Desenvolvimento do inquérito

Um questionário destinado a esclarecer as práticas relacionadas à sedação e à analgesia nas UTI pediátricas brasileiras foi desenvolvido pelos pesquisadores, baseado em revisão da literatura, utilizando-se os bancos de dados PubMed^®^, EMBASE e *Scientific Electronic Library Online* (SciELO), no período de janeiro de 2010 a janeiro de 2017, com as palavras chave “ *sedation and analgesia and children* ”, “ *delirium and children* ”, “ *sedation and (guidelines or protocol) and children* ”, “ *analgesia and (guidelines or protocol) and children* ”.

A pesquisa foi testada e validada entre cinco intensivistas pediátricos de diferentes centros brasileiros, utilizando-se ferramenta de sensibilidade clínica em forma de questionário. Seguiu-se o retorno sobre cada questão da pesquisa, abordando a abrangência e redação, a presença de itens redundantes ou inapropriados, e se o questionário cumpria os objetivos da pesquisa. As respostas dos testes-piloto não foram incluídas nos principais resultados da pesquisa relatada. O inquérito foi distribuído para pediatras intensivistas cadastrados no banco de dados da Associação de Medicina Intensiva Brasileira (AMIB). O estudo foi aprovado pelo Comitê de Ética em Pesquisa da Associação Congregação de Santa Catarina, parecer 3.077.035, CAAE: 02880918.1.0000.9007. A fase de coleta de dados via correio eletrônico ocorreu de junho a novembro de 2017.

### Características do inquérito

O inquérito consistiu de 14 questões de múltipla escolha, sendo que as primeiras seis questões eram sobre as características demográficas dos médicos e locais de trabalho. As oito questões restantes eram especificamente sobre as práticas diárias relacionadas à sedação e à analgesia, a utilização de protocolos e o rastreamento do *delirium* em suas respectivas UTI pediátricas.

### Análise estatística

Foram realizadas análises descritivas dos dados obtidos por meio de cálculos de frequências simples e proporções utilizando-se o software R, versão 3.5.0.

## RESULTADOS

A amostra foi composta por 61 médicos pediatras intensivistas que responderam ao inquérito, dos 373 e-mails enviados (taxa de resposta de 16,3%). Após a análise inicial das respostas, cinco médicos foram excluídos, por atuarem exclusivamente em UTI neonatais. Na tabela 1 , é possível observar as caraterísticas demográficas e descritivas dos locais de trabalho dos participantes. A maioria dos médicos intensivistas que respondeu ao questionário era da Região Sudeste (57,2%), e 73,2% trabalhavam em UTI pediátricas com no máximo 15 leitos disponíveis. Do ponto de vista da gestão, 46,5% trabalhavam em hospitais públicos, estando 28,6% deles sob administração direta do Estado e 17,9% sob administração indireta, e 41,1% dos profissionais trabalhavam em hospitais privados. Dentre os médicos que responderam ao inquérito, 64,3% trabalham em hospitais que ofereciam programa de residência médica em medicina intensiva pediátrica.

**Tabela 1:** Características descritivas e demográficas dos hospitais e dos pediatras participantes da pesquisa

Características	
Região	
Norte	1,8
Nordeste	14,3
Centro Oeste	14,3
Sudeste	57,0
Sul	12,6
Tipo de UTI	
UTI pediátrica	41 (73,2)
UTI mista (pediátrica + neonatal)	6 (10,7)
UTI cardiológica	3 (5,4)
Outras	6 (10,7)
Número de leitos	
<10	19 (33,9)
10-15	22 (39,3)
16-20	10 (17,9)
>20	5 (8,9)
Gestão do hospital	
Público (administração direta)	16 (28,6)
Público (administração indireta)	10 (17,9)
Privado	23 (41,1)
Misto	5 (8,9)
Não respondeu	2 (3,5)
Programa de residência médica	
Sim	36 (64,3)
Não	20 (35,7)
Pacientes em VM na UTI, %	
<25	8 (14,3)
25-50	22 (39,3)
51-75	18 (32,1)
>75	8 (14,3)
Tempo de residência médica, anos	
1	6 (10,7)
2	8 (14,3)
3	13 (23,2)
4	23 (41,1)
>4	6 (10,7)
Tempo de trabalho em UTI, anos	
<3	12 (21,4)
3-6	11 (19,7)
6-9	5 (8,9)
9-12	8 (14,3)
>12	20 (35,7)

Resultados expressos por % ou n (%). UTI: unidade de terapia intensiva; VM: ventilação mecânica.

A tabela 1 mostra as características dos médicos relacionadas ao tempo de formação. A maioria dos médicos (51,8%) cumpriu 4 ou mais anos de residência médica. Em relação ao tempo de prática clínica em UTI pediátrica, observamos distribuição bimodal nos extremos, sendo que 41% responderam que tinham 6 anos ou menos de experiência profissional, e 35,7% tinham mais que 12 anos de experiência profissional.

Como as práticas de sedação e analgesia estão relacionadas principalmente aos pacientes submetidos ao uso de VM, uma das questões era relacionada à percentagem de pacientes, na média diária, que recebiam VM. As respostas foram que 39,3% apresentavam de 25 a 50% dos pacientes sob VM e 32,1% apresentavam de 50 a 75% dos pacientes sob VM, totalizando 71,4%, que ficavam entre 25% e 75% de pacientes sob VM diariamente.

Especificamente em relação à prática clínica, a maioria (87,5%) dos respondedores utilizava escalas de sedação e analgesia em suas unidades, embora apenas 57,1% dos locais de trabalho tivessem protocolo formal. As escalas mais utilizadas foram a de Ramsay (53,6%) e a de agitação e sedação de Richmond (RASS - *Richmond Agitation-Sedation Scale* ) (26,8%) ( Figura 1 ). Nos locais que utilizavam protocolos, a maioria era gerenciada por médicos (53,6%). Em contraste, 48,2% dos médicos não utilizavam escores de rastreamento de *delirium* , tendo sido a *Cornell Asssessment of Pediatric Delirium* (CAP-D) (23,2%) a mais utilizada ( Figura 2 ).

**Figura 1:** Escalas de avaliação de sedação e analgesia utilizadas pelos participantes da pesquisa EVA: escala visual analógica; RASS: escala de agitação e sedação de Richmond.

**Figura 2:** Escalas de avaliação de *delirium* utilizadas pelos participantes da pesquisa p-CAM: *Pediatric Confusion Assessment Method for the Intensive Care Unit* ; PAED: *Pediatric Anesthesia Emergence Delirium Scale* ; CAP-D: *Cornell Asssessment of Pediatric Delirium.*

Em relação às outras práticas avaliadas no estudo, o uso de intervenções não farmacológicas foi frequentemente ou sempre feito por apenas 23,2% dos respondedores. Referente à participação dos pais com informações sobre a presença de dor ou agitação, as respostas foram heterogêneas, e 44,6% responderam que existia uma participação mais efetiva dos pais nesse processo ( Figura 3 ). Sobre a prática diária de interrupção de sedação em pacientes sob VM, a maioria (85,7%) respondeu que não utilizava essa prática na UTI pediátrica ( Figura 3 ).

**Figura 3:** Outras intervenções relacionadas à prática diária de sedação, analgesia e *delirium* UTI: unidade de tereapia intensiva.

## DISCUSSÃO

Este é o primeiro estudo que verificou o uso de ferramentas clínicas para avaliação de sedação, analgesia e prevenção do *delirium* entre médicos pediatras intensivistas no Brasil. O estudo mostra que a dor e a sedação são rotineiramente avaliados na prática clínica dos intensivistas pediátricos, que informaram seguir diretrizes e protocolos de sedoanalgesia em suas unidades. A minoria (12,5%) não utiliza ou não conhece escores de sedação/analgesia. Esses dados revelam boa adesão à utilização de ferramentas de avaliação da sedoanalgesia nos pacientes. Comparando com a mesma prática em outros países, Garcia Guerra et al.,^5^ reportam que 84% dos intensivistas pediátricos no Canadá utilizam escores de sedação/analgesia em suas unidades, sendo as escalas COMFORT (41%) e COMFORT-B (15%) as mais utilizadas. Kudchadkar et al.,^6^ em estudo internacional (embora 70% dos participantes fossem da América do Norte), mostraram que, mesmo que 70% dos respondedores de um questionário tenham dito ter escores de sedação em suas UTI pediátricas, apenas 42% os utilizavam rotineiramente para determinar as metas de atendimento ao paciente. Um dos principais objetivos no desenvolvimento de protocolos da avaliação de sedação e analgesia é a utilização de ferramentas clínicas validadas para uso em pediatria e que permitam o uso racional de medidas farmacológicas e não farmacológicas para o conforto do paciente.

Em relação à avaliação do *delirium* pelos pediatras que responderam ao inquérito, 51,8% utilizavam alguma ferramenta de avaliação, sendo a CAP-D a mais usada. Delineia-se aqui importante lacuna de avaliação do paciente entre os participantes da pesquisa. Em estudo de Kudchadkar et al.,^6^ 71% dos entrevistados relataram que sua unidade não realiza triagem de *delirium* de rotina, e apenas 2% relataram que o rastreamento de *delirium* é feito em todas as crianças pelo menos uma vez por turno.^6^ Avaliação de *delirium* também é incomum nas UTI pediátricas canadenses.^5^ O *delirium* tem sido cada vez mais reconhecido como complicação frequente dos cuidados intensivos e claramente associado a evoluções negativas, incluindo a mortalidade. Diretrizes de sedação em adultos recomendam seu monitoramento de rotina.^7 , 8^ No entanto, há uma falta de estudos pediátricos de alta qualidade, e as estimativas atuais de prevalência variam de 4,5% a 28%.^9^ Além disso, a melhor abordagem para prevenção, detecção e manejo do *delirium* pediátrico não é clara. Qualquer estratégia de tratamento, no entanto, depende do reconhecimento e do monitoramento de rotina como o primeiro passo necessário. Infelizmente, os sintomas do *delirium* pediátrico são frequentemente tratados com mais sedação, gerando um ciclo vicioso contribuindo para o aumento da morbidade e da mortalidade.^10^

A prática de interrupção diária da sedoanalgesia não é realizada na maioria das UTI pediátricas. Trata-se de prática mais comum em pacientes adultos, sem evidências de benefício na interrupção diária da sedação em pediatria. Em estudo multicêntrico randomizado controlado, mostrou-se que a interrupção diária da sedação em crianças sob VM, associada ao uso de protocolos de sedação, não melhorou o desfecho clínico e esteve associada ao aumento da mortalidade em comparação com apenas o uso de protocolo de sedação.^11^ Por outro lado, Zimmerman et al., em carta ao editor, criticam esse estudo e sugerem que novas pesquisas sobre a prática de interrupção diária de sedação devam ser realizadas, com abordagem multidisciplinar.^12^ Há um campo aberto para estudos sobre esse assunto em pediatria.

Apesar da grande variação nas respostas, os pais tiveram alguma participação no gerenciamento da dor/agitação do paciente na amostra pesquisada. Cada vez mais os pais têm participado ativamente das decisões relativas ao quadro clínico dos filhos internados em UTI pediátrica. Faltam estudos de qualidade que comprovem os possíveis benefícios dessa crescente participação no gerenciamento da dor e do *delirium* .

O estudo mostra que medidas de conforto não farmacológicas foram comumente utilizadas na amostra pesquisada. A promoção de um ambiente com luminosidade e sonoridade adequados, para evitar o estresse e promover o sono, é importantes em uma unidade de cuidados críticos. Em revisão sistemática, Kudchadkar et al.,^13^ consideram que “embora essas abordagens preventivas e de tratamento possam ser consideradas simples e de baixo custo de implementação, há ainda uma grande mudança cultural que deve ocorrer na comunidade de cuidados intensivos pediátricos”.^13^ Evidências científicas e clínicas são imperativas para demonstrar que a otimização do sono em crianças gravemente doentes pode reduzir a morbidade, por meio da diminuição de medicamentos sedativos, da neuroinflamação e do tempo de permanência hospitalar. Outras intervenções não farmacológicas adicionais podem ser implementadas pelo uso de chupeta, música, televisão e dispositivos eletrônicos com jogos (tablets), entre outros.

Deve-se destacar a importância da iniciativa *ICU Liberation* da *Society of Critical Care Medicine* (SCCM) para liberar os pacientes dos efeitos adversos da dor, da agitação e do *delirium* na UTI.^14^ As diretrizes da *ICU Liberation* e o pacote de medidas ABCDEF são recursos vitais para avaliar, tratar e prevenir a dor, a agitação e o *delirium* .^14 , 15^ A iniciativa também está focada em estratégias de mobilidade precoce, que podem ajudar a reduzir o risco de consequências a longo prazo de uma internação na UTI.

Este estudo apresenta limitações. A taxa de resposta ao questionário foi baixa (16,3%) em uma comunidade médica que é pequena, se comparada aos intensivistas adultos, reduzindo a força estatística do estudo. A amostra tem viés de seleção, pois foram convidados a participar do inquérito apenas intensivistas pediátricos com cadastro na AMIB, titulados ou não, podendo não representar a população geral de intensivistas pediátricos no Brasil. A maioria dos respondedores foi da Região Sudeste do Brasil, principalmente do estado de São Paulo, impedindo a generalização dos resultados para todo do país.

## CONCLUSÃO

Este estudo destaca a heterogeneidade nas práticas de avaliação da sedação/analgesia e insuficiência do rastreamento de *delirium* em uma amostra de intensivistas pediátricos brasileiros. Existem inúmeras oportunidades para futuros estudos, melhorias de qualidade, desenvolvimento de protocolos e intervenções terapêuticas em população pediátrica em ativo desenvolvimento neurocognitivo.
